# Can Satellites Predict Yield? Ensemble Machine Learning and Statistical Analysis of Sentinel-2 Imagery for Processing Tomato Yield Prediction

**DOI:** 10.3390/s23052586

**Published:** 2023-02-26

**Authors:** Nicoleta Darra, Borja Espejo-Garcia, Aikaterini Kasimati, Olga Kriezi, Emmanouil Psomiadis, Spyros Fountas

**Affiliations:** 1 Laboratory of Agricultural Machinery, Department of Natural Resources Management and Agricultural Engineering, Agricultural University of Athens, 75 Iera Odos Str., 11855 Athens, Greece; 2 Laboratory of Mineralogy and Geology, Department of Natural Resources Management and Agricultural Engineering, School of Environment and Agricultural Engineering, Agricultural University of Athens, 75 Iera Odos Str., Votanikos, 11855 Athens, Greece

**Keywords:** Sentinel-2, AutoML, NDVI, PVI, WDVI, SAVI, RVI, yield prediction, processing tomato

## Abstract

In this paper, we propose an innovative approach for robust prediction of processing tomato yield using open-source AutoML techniques and statistical analysis. Sentinel-2 satellite imagery was deployed to obtain values of five (5) selected vegetation indices (VIs) during the growing season of 2021 (April to September) at 5-day intervals. Actual recorded yields were collected across 108 fields, corresponding to a total area of 410.10 ha of processing tomato in central Greece, to assess the performance of Vis at different temporal scales. In addition, VIs were connected with the crop phenology to establish the annual dynamics of the crop. The highest Pearson coefficient (r) values occurred during a period of 80 to 90 days, indicating the strong relationship between the VIs and the yield. Specifically, RVI presented the highest correlation values of the growing season at 80 (r = 0.72) and 90 days (r = 0.75), while NDVI performed better at 85 days (r = 0.72). This output was confirmed by the AutoML technique, which also indicated the highest performance of the VIs during the same period, with the values of the adjusted R^2^ ranging from 0.60 to 0.72. The most precise results were obtained with the combination of ARD regression and SVR, which was the most successful combination for building an ensemble (adj. R^2^ = 0.67 ± 0.02).

## 1. Introduction

Yield mapping is the process of collecting and analyzing field-level crop yield data, offering several benefits, including improved efficiency, increased profitability, better resource management, and improved food security [1,2]. It is a valuable tool for farmers because it allows them to monitor and optimize the productivity of their fields and make informed decisions about management practices. The more information they have about the yield of each crop and field on their farm, the better they can assess the impact of decisions made during the growing season and improve management decisions for subsequent seasons [3]. This interest in yield monitoring systems has triggered the development and adoption of smart farming methods, as they provide a large amount of information on key agricultural parameters from the field level to larger geographic areas. Through the use of a range of advanced technologies and data analysis techniques, smart farming can optimize crop management practices, especially in intensive cropping systems such as those of processing tomato (*Lycopersicon esculentum*).

Considering that global trade of processed tomato products is worth more than USD 7.5 billion annually and the exports of finished products reached 6.9 million tons in the 2021/2022 period, as reported by the World Processing Tomato Council (WPTC) [4], the socioeconomic importance of this crop at the international level is obvious. In Greece, the total area under processing tomato in 2018 was 4102.20 hectares, corresponding to an average yield of 71 tons per hectare, which varies depending on the hybrid, weather conditions, soil, and cultivation practices [5]. Although processing tomato is a dynamic crop for the agricultural economy, climatic uncertainties and increased demands on agricultural water sources threaten the sustainability of production. Increasing demand for higher value-added products such as canned tomatoes, passata, tomato sauces, and organic products has created a need to improve tomato yield and quality.

Among smart farming data streaming technologies [6,7], the most common approaches assume that yield is correlated with photosynthetically active biomass and other crop parameters at an important phenological stage of the crop. As a result, a variety of sensors and vegetation indices (VIs) have been used and reported to correlate well with a range of vegetation parameters, including the ability to predict yield [8,9]. While considerable research efforts have been made in the area of smart farming systems, only a limited number of studies has been focused on processing tomato crop. In the past, Fortes et al. [10] conducted field research at two commercial tomato processing plants and used measurements of apparent electrical conductivity and NDVI measurements to estimate processing tomato yield. Using two geostatistical methods, an NDVI map was developed to predict the yield of processing tomatoes [11]. Gianquinto et al. [12,13] investigated the stability of several vegetation indices (the main VIs considered in a previous study) in assessing the canopy reflectance of a processing tomato crop against the main plant traits described as sources of variation (chlorophyll and N content; N supply), including yield prediction.

In recent years, following the tremendous advances made in access to Earth observation (EO) products, data, and services, several studies have focused on the use of satellite imagery to estimate crop variables. Campillo et al. [14] used satellite imagery to distinguish productivity zones in processing tomato. Spatial variability was examined using various NDVI images from the Sentinel-2 satellite. They served as a control point to measure the evolution of the crop throughout the crop cycle and define different productivity zones [14,15]. According to Vaglio Laurin et al. [16], Sentinel-2 has proven to be a valuable tool for monitoring growth and crop damage in a given crop. In their study, early tomato maps based on Sentinel-2 data had an accuracy of >80% for users in seven out of nine cases and >80% in five out of nine cases, with differences due to the different agricultural characteristics and environmental heterogeneity of the study areas. Finally, a multilevel model for tomato yield prediction was developed by Psiroukis et al. [17] using spectral indices from Sentinel-2 imagery.

A widely used method to examine the relationship between two or more variables of interest is the combination of statistical and regression analyses, including descriptive statistics. Traditionally, Pearson correlation is used to determine the spatial relationship between canopy NDVI and crop yield, and linear and multivariate regression models are used to determine field-wide production. As computer power has increased significantly in recent decades, more sophisticated machine learning approaches have been developed to predict crop yield [11,18]. As early as 2005, Koller and Upadhyaya [19] developed a neural network model to predict processing tomato yield based on the conservation of mass using daily LAI values, along with PAR data and other crop-specific parameters. Their results showed that although the actual and predicted yield maps did not have a very high correlation, the two maps had similar yield patterns. Although the application of machine learning in agriculture is currently occurring at a rapid and effective pace [20], widespread use of these techniques remains a challenge. Their successful application is not effortless and still relies heavily on specialized human resources [21]. They typically require the extensive involvement of experts working iteratively to develop the most appropriate machine learning pipeline. One solution to this problem may be automated machine learning (AutoML), which offers the opportunity to improve this task and save time and human resources by automating the time-consuming, iterative tasks of developing machine learning pipelines. For instance, model selection and optimal tuning of hyperparameters can be achieved automatically. AutoML systems are meta-level machine learning algorithms that find high-performance pipeline designs based on previous individual machine learning solutions [22,23]. These systems automatically evaluate alternative pipeline topologies and attempt to iteratively improve performance for a given task and dataset [24]. In addition, engineers can focus on the final implementation details to deploy AutoML-provided solutions. This workflow results in better models being delivered in less time. At the same time, these workflows can provide domain researchers with a new understanding of how their input data, such as NDVI, interact with the yield prediction models. Recently, Pelta et al. [25] used NDVI from Sentinel-2 and Landsat-8, meteorological data, and artificial intelligence to predict the seasonal crop coefficient for processing tomatoes.

While previous research has explored various correlation and regression models between VIs and crop production, as well as machine learning techniques for estimating crop yield, AutoML, as described above, has not been widely explored. In the agricultural field, the use of AutoML techniques has only been recorded for time series processing and analysis of proximal and satellite imagery [26,27], weed identification [28], and prediction of quality attributes in grapes [29]. To the best of our knowledge, the use of ensemble regressors in yield prediction is not well studied. Since finding the best ensembles could lead to a combinatorial explosion, AutoML was explored as a methodology to address this issue, with promising results. In this paper, we propose a novel approach for robust yield prediction of processing tomatoes using five different VIs (NDVI, PVI, RVI, WDVI, and SAVI) and ensembles of machine learning models automatically found by AutoML techniques. VIs were obtained from Sentinel-2 imagery using non-destructive methods during the 2021 growing season. This study also provides some clear results about which are the most effective growth stages and VIs for yield prediction.

## 2. Materials and Methods

### 2.1. Study Area

**Figure 1:**
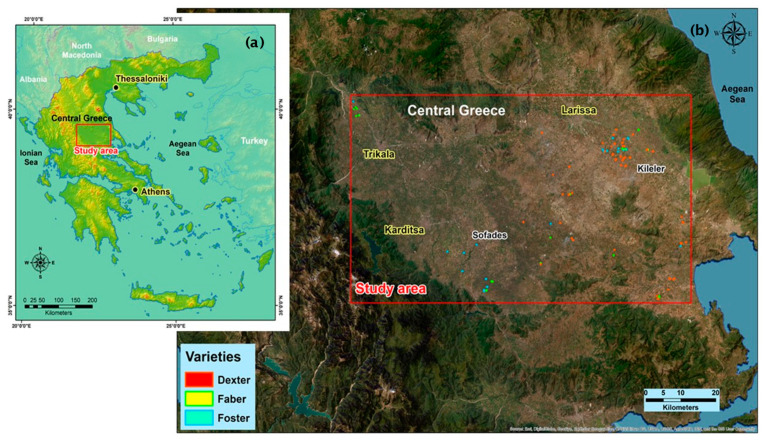
The study area: (**a**) map of Greece and the location of the study area; (**b**) the distribution of fields in the study area.

This study took place in the wider area of central Greece (Figure 1a), where 108 fields corresponding to a total area of 410.10 ha of processing tomato were selected as pilot fields (extent of E: 22°13′20″ N: 39°42′40″, E: 23°6′40″ N: 39°10′40″). The experimental process included fields of three different hybrids. Specifically, the Dexter hybrid corresponded to 62 fields in an area of 242.40 ha, the Faber hybrid corresponded to 20 fields in an area of 66.80 ha, and the Foster hybrid corresponded to 26 fields in an area of 100.90 hectares (Figure 1b). All pilot fields ranged in size from 1 to 14 ha and were planted in rows with an average row spacing of 0.4–0.6 m, which corresponds to the extensive cropping system commonly used in the region. The planting date of the pilot fields was different and ranged between mid-April and mid-May 2021, while harvesting was completed in all fields in early September. The pilot fields were digitized using georeferenced layers (.kml) of each field’s boundaries, which were collected by agronomists in May 2021. An example of the vector layers of field boundaries collected during these field measurements is presented in Figure 1. Finally, the actual yield of each pilot field was recorded directly by the farmers under the supervision of agronomists, and the respective total yield values were included in the dataset.

### 2.2. Satellite Imagery

The remote sensing data used in this study are 10 × 10 m^2^ resolution multispectral images acquired by the multispectral imager (MSI) sensor on ESA’s Sentinel-2 satellite platforms. Atmospherically corrected imagery (level 2A, providing values for reflectance at the bottom of the atmosphere) from the Sentinel-2A/B satellite in cartographic geometry (UTM/WGS84 projection) was acquired free of charge from the ESA portal (https://scihub.copernicus.eu/, accessed on 13 October 2022). Satellite imagery data were collected at 5-day intervals, but the sample size was not always constant due to total cloud cover (Table 1). For each date, a set of three (3) satellite images was downloaded to cover the entire study area.

Preprocessing of the data included resampling the images to 10 m because the spectral bands of Sentinel-2 operate at different spatial resolutions of 10 m (4 bands: B2, B3, B4, and B8), 20 m (6 bands: B5, B6, B7, B8A, B11, and B12), and 60 m (3 bands: B1, B9, and B10). On the days when heavy cloud cover occurred and the experimental fields of interest were not visible, the corresponding values were removed from the dataset to ensure the validity of the data.

To evaluate the satellite systems and their relationship to yield, five VIs, namely the normalized difference vegetation index (NDVI), weighted difference vegetation index (WDVI), soil-adjusted vegetation index (SAVI), ratio vegetation index (RVI), and perpendicular vegetation index (PVI), were calculated for each date via SNAP software (Sentinel Application Platform—ESA Sentinels Application Platform v6.0.4), which is provided free of charge and accessible to everyone as part of the European Copernicus project. As a result, VI raster datasets of the whole area were created by iterating the VI formulas over all satellite image pixels.

The second step of data preprocessing involved the mosaicking of the individual images of each survey date into a single raster dataset of the entire study area using ArcGIS software (Environmental Systems Research Institute, Redlands, CA, USA). Once the total number of images was determined, an additional manual filtering step was performed to ensure that each generated mosaic consisted solely of high-quality and cloud-free data from the pilot fields. Given the small size of the fields, pixels outside the pilot farm boundaries were also selected and masked. For each date, a mean VI value was extracted from each field using the zonal statistics tool of the ArcGIS software. The results were recorded in Excel spreadsheets with 108 NDVI, RVI, PVI, SAVI, and WDVI values for all the measurement dates acquired.

### 2.3. VIs

**Figure 2:**
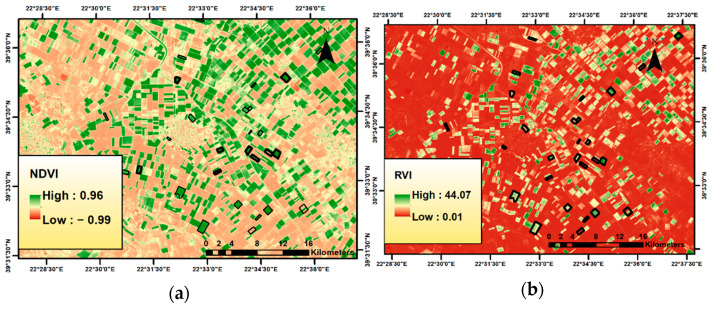
A segment of the study area in the wider area of Larissa: (**a**) NDVI; (**b**) RVI.

The most widely used vegetation indices are the NDVI (normalized difference vegetation index) and the weighted difference vegetation index (WDVI). Other commonly used VIs are the soil-adjusted vegetation index (SAVI), the ratio vegetation index or the simple ratio vegetation index (RVI), and the perpendicular vegetation index (PVI). For the calculation of the VIs, bands 4 and 8 of Sentinel-2 were used, which correspond to the RED and NIR spectrum, respectively. The equations used for the estimation VIs are presented below (Table 2):

NDVI is the most commonly used vegetation index and has found various applications. The result of NDVI calculation is an image with a continuum of pixel values ranging from −1 to 1 (Figure 2a). The NDVI varies from a minimum at bare soil reflectance to a maximum for a fully developed canopy with a value slightly less than one [30]. Healthy photosynthetic vegetation is related to higher positive values; on the other hand, stressed vegetation or even bare soil is related to lower values, especially <0.2 [36,37]. In the processing tomato crop, NDVI values are reported to have good correlation with several vegetation parameters, including the ability to predict yield [11].

The same spectral bands were used for RVI (ratio vegetation index or simple ratio vegetation index), which is recorded to improve both saturation in high vegetation and sensitivity to the soil in low vegetation compared with NDVI [38]. It was introduced by Pearson and Miller [34]) and is based on the contrast between the visible red and far-infrared bands of electromagnetic radiation for the pixels corresponding to vegetation [34]. High values of the index are mainly attributed to healthy vegetation and result from the combination of its low reflectance value for the red and the high reflectance it presents in the near-infrared band. Its value range is from 0 to more than 30, with healthy vegetation usually presenting values of 2 to 8 (Figure 2b). 

**Figure 3:**
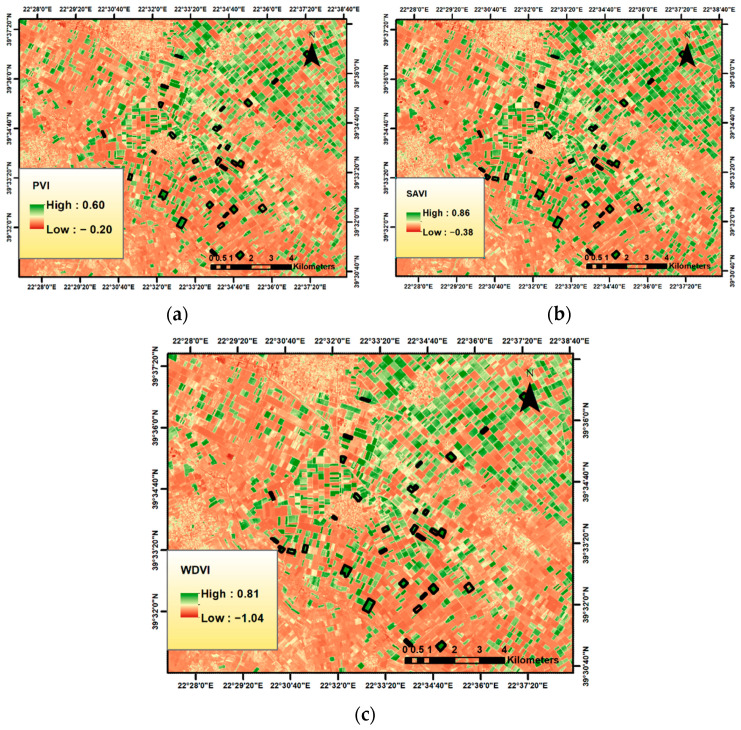
A segment of the study area in the wider area of Larissa: (**a**) PVI; (**b**) SAVI; (**c**) WDVI.

Richardson and Wiegand [33] approached the problem of variable soil brightness by developing the perpendicular vegetation index (PVI), which attempts to eliminate differences in soil background (Figure 3a) [33]. It can be computed as a spectral indicator of plant development or biomass accumulation and cannot be considered to be independent of soil brightness. While it is effective in removing soil brightness effects for bare soil, it quickly becomes more sensitive as the canopy develops. A PVI value of 0 indicates bare soil, whereas negative values indicate water and positive values indicate vegetation. It is less sensitive to the atmosphere but is considered sensitive to the reflectivity and brightness of the ground, especially in cases with low vegetation cover.

The weakness presented by PVI regarding the assumption that there will be only one soil type under vegetation is addressed by the soil-adjusted vegetation index (SAVI), which was proposed by Huete [35] and is a hybrid of NDVI and PVI. The originality of this index lies in the establishment of a simple model that permits an adequate description of the soil–vegetation system [39]. SAVI (Figure 3b) also attempts to eliminate soil background effects; however, it is much less sensitive to changes in the background caused by soil color or surface soil moisture content than the ratio vegetation index [40]. Qi et al. [41] showed that the adjustment factor (L) is not a constant but a function that varies inversely with the amount of vegetation present. Generally, it is best applied to soils with sparse vegetation, and its range of desired values is the same as that of NDVI [42].

The WDVI (weighted difference vegetation index) was introduced by Clevers et al. [31] in 1989. WDVI (Figure 3c) has been used to overcome high PVI values due to a bright soil background. This index is also based on distance, and it assumes that the ratio between NIR and the red reflectance of bare soil is constant [43]. The WDVI concept was developed in order to correct for the influence of soil background, but it is quite sensitive to atmospheric conditions. It is mathematically simpler than the rest of the indicators but with an infinite range of desired values [32].

### 2.4. Statistical Analysis

Once actual yield data were collected, statistical analysis was performed to evaluate and establish relationships between yield and VIs using XLSTAT software (Addinsoft, www.xlstat.com, accessed on 1 November 2022). A crucial step prior to the analysis was the removal of artifacts within the data, which may appear as extraneous data points that fall outside the general range of the dataset (referred to as outliers). Since their presence indicates a possible measurement error, they were removed in the preprocessing phase. Descriptive statistics and graphs were generated, and linear correlation was investigated to find relationships between the yield data and the satellite-derived VIs. Specifically, Pearson’s correlation coefficient (r) was used to assess the spatial similarity between the recorded yield and the VI variables. Then, AutoML was deployed to further evaluate the relationships between the yield and the respective VIs.

### 2.5. Automated Machine Learning

Machine learning techniques can sometimes improve the modeling capacity of traditional statistical techniques. However, the availability of hundreds of machine learning algorithms makes choosing the right one a major challenge. Moreover, each of these algorithms has multiple hyperparameters that must be fine-tuned by trial and error. This means that there is no a priori knowledge about the best fit, and they are not optimized during the training process; for example, the number of trees for Random forests and AdaBoost, the splitting criterion (e.g., Gini, entropy, etc.) for all tree-based methods, and the sensitivity to outliers for robust linear regression methods such as Theil-Sen or Huber. Therefore, it is critical to automate this process through AutoML and focus on gaining scientific knowledge as to which is the best time in a season to predict yield.

**Figure 4:**
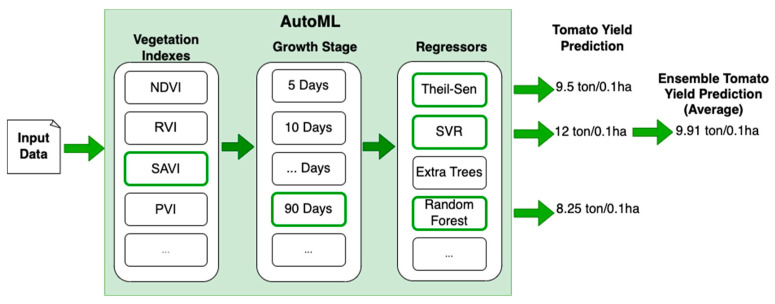
The use of AutoML for selection of the best combination of inputs (vegetation index and growth stage) and creating an ensemble of regression models is proposed as the methodology.

AutoML is a field of research that has become increasingly popular over the last few years [44]. Different domains, such as image recognition [45] and time series processing [46], take advantage of this technique. Moreover, some specific subfields of AutoML, such as neural architecture search (NAS), have arisen to optimize the search for some specific hyperparameters in the design of neural architectures (e.g., number of layers, activation function, etc.). However, there are still some open concerns [47] because (i) finding the best hyperparameters can still be too computationally expensive and (ii) AutoML adds a new layer of complexity/abstraction that can make the interpretability of the model decisions harder. On the other hand, more studies are arising around this topic; therefore, agriculture, specifically yield prediction, should be used to evaluate the current state of the technologies implementing AutoML techniques. In this study, AutoML was also used to create ensembles based on the best models found during the optimization process. Ensemble models aim to improve the performance of machine learning models by combining several of them [48]. In the case of regression, the mean of the predictions of the models with the best performance is used as the final prediction (see Figure 4). An ensemble can be composed of endless models; however the larger the amount, the higher the computational requirements. Therefore, in this study, ensembles of up to 3 regressors were evaluated.

Although AutoML can use any type of machine learning algorithm, AutoML was studied in this paper to extend our previous work on AutoML without ensembles [29]. Linear and nonlinear regression algorithms were used, including ordinary least square, automatic relevance determination regression, Theil-Sen, and Huber regression models, as well as decision-tree-based algorithms:

- Ordinary least squares (OLS): the most common estimation method for computing linear regression models, which can be found in related work, e.g., Prasetyo et al. [49];
- Automatic relevance determination (ARD) regression: compared to the OLS estimator, the coefficient weights are shifted slightly toward zeros, which stabilizes them [50];
- Theil-Sen estimator method: the most popular non-parametric technique for estimating a linear trend, making no assumptions about the underlying distribution of the input data [51];
- Huber regression: this model is aware of the possibility of outliers in a dataset and assigns them less weight than other samples, in contrast to Theil-Sen, which ignores them [52];
- Decision trees: this method uses a non-parametric learning approach. Its main advantage is that it can be visualized to better understand why the classifier made a particular decision.

To improve the predictive power of the model, in this study, we also evaluated several ensemble methods based on decision trees, such as AdaBoost, fandom forests, and extra trees. These methods combine the predictions of multiple tree-based models to make more accurate predictions than the individual models. Specifically, these ensemble methods start with a decision tree and then use boosting or bootstrap aggregation to reduce its variance and bias (bagging). It is important to remark that these tree ensembles are different from the ensemble of models that are built on top of the system. This means that the final ensemble used to compute the regression can be composed of three tree ensembles (e.g., two random forests and one AdaBoost).

- AdaBoost: The AdaBoost algorithm (adaptive boosting) uses an ensemble learning technique known as boosting, whereby a decision tree is retrained several times, with greater consideration given to data samples for which the regression is imprecise [53];
- Random Forest: A supervised learning approach in which the ensemble learning method is used for regression. In this approach, numerous decision tree regressors are combined into a single model trained for many data samples collected on the input characteristic (in this case, NDVI) using the bootstrap sampling method [54];
- Extremely Randomized Trees: Extra trees is similar to random forest in that it combines predictions from many decision trees, but instead of bootstrap sampling, it uses the entire original input sample [55].

### 2.6. AutoML Software

In this study, the auto-sklearn framework [22] was used to implement the AutoML pipeline. This means that three main techniques were used. First, Bayesian optimization was used as the global optimization algorithm. Since finding the best regressor and its hyperparameters is a non-convex, computationally expensive problem, the Bayes theorem can be used to direct an efficient and effective search of an optimal hyperparameter configuration [56]. Secondly, a metalearning step was used to warm start the Bayesian optimization procedure, which resulted in a considerable boost in efficiency. In the case of auto-sklearn, the metalearning approach used an offline phase to learn the best initialization configurations along 140 datasets from the OpenML [57] repository. Thirdly, auto-sklearn implements an ensemble building technique whereby the most suitable models are combined to boost the prediction performance.

## 3. Results

### 3.1. Statistical Analysis

Before performing the correlation analysis, basic statistics were calculated to examine the data. According to the descriptive statistics of the actual yield (Table 3), the values ranged from 40 to 135 t/ha. The mean values ranged from 86.68 to 98 t/ha depending on the hybrid. These values are considered high, considering the national average production of processing tomatoes (71 t/ha, Ministry of Rural Development and Food). The coefficient of variance (CV) ranged from 20.42% to 25.13%, which is relatively high, considering the total number of fields. Overall, the Dexter hybrid had the highest yield average, while the lowest value was observed in the Foster hybrid.

**Figure 5:**
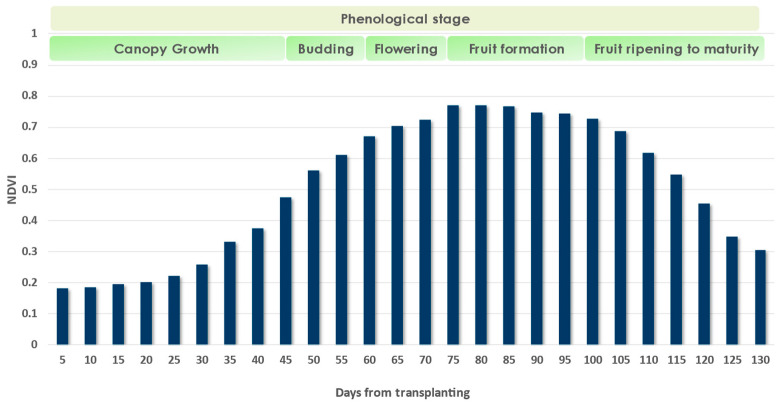
Annual NDVI dynamics and the respective phenological stages of the processing tomato crop.

It was found that the highest NDVI values were recorded 75 to 80 days after transplanting (Figure 5). In the early stages, NDVI values were low, which is to be expected, considering that in row crops, the soil was clearly visible in the remotely sensed images. Full canopy cover and flowering were recorded in June (60–75 days after transplanting), while the tomato formation phase occurred in July, depending on the transplanting date. This is consistent with a previous study [58] in which phenological monitoring was performed for the period of 2016–2021 using NDVI values from Sentinel-2 imagery. Based on the reported NDVI values, Figure 5 shows the NDVI dynamics and the corresponding phenological stages of the crop.

**Figure 6:**
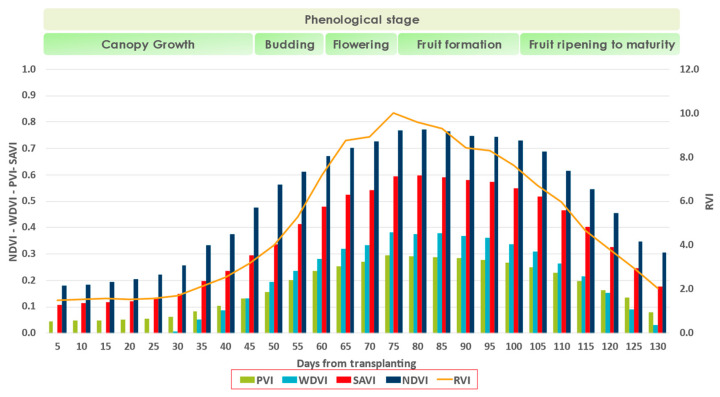
The mean values of the five VIs: PVI (green); WDVI (light blue); SAVI (red); NDVI (blue); RVI (orange), which has different range of values and is incorporated in the secondary axes.

Not surprisingly, based on the mean values of all five VIs (NDVI, PVI, WDVI, SAVI, and RVI), progressive canopy growth is observed (Figure 6). In the early stages, the influence of soil is strong due to the low canopy cover. There seems to be a positive trend that peaks at 80 days and is negative in the last stages of the crop.

Statistical analysis of the dataset shows a positive linear relationship between VIs and yield throughout the season. In the first 45 days, the relationships were weak (Pearson correlation coefficient (r) < 0.30), indicating poor predictive ability. However, in the following period, the Pearson correlation coefficient increased for all VIs, reaching the highest values in the period from 75 to 95 days after transplanting, depending on the VI and hybrid, as shown in Table 4, Table 5, Table 6 and Table 7. For the cultivar Dexter (Table 4), the dataset includes 62 fields corresponding to a range of 50 to 62 observations for each date depending on the cloud cover. RVI had the highest values of Pearson coefficient at 80 (r = 0.71, *p* < 0.05) and 90 days (r = 0.75, *p* < 0.05), while NDVI had the highest values at 85 days (r = 0.718, *p* < 0.05). SAVI and PVI performed well at 90 days, while the highest correlations were shown at 95 days (r = 0.72, *p* < 0.05) by WDVI.

For the Faber cultivar, the dataset includes 20 fields corresponding to a range of 17 to 20 observations for each date depending on the cloud cover. The r value of the VI datasets ranged from 0.58 to 0.90 (Table 5), and satisfactory values appeared after 75 days of the growing season. The highest correlation at 75 (r = 0.82, *p* < 0.05) and 85 days (r = 0.85, *p* < 0.05) was obtained by NDVI, whereas RVI, SAVI, PVI, and WDVI values obtained during the same period presented r values ranging from 0.58 to 0.81. SAVI appeared to be better at 90 days (r = 0.90, *p* < 0.05) and PVI at 80 days (r = 0.82, *p* < 0.05).

For the Foster hybrid, the dataset includes 26 fields corresponding to a range of 23 to 26 observations for each date depending on the cloud cover. RVI values were higher throughout the 75- to 90-day post-planting period (Table 6). In particular, during the 75–85-day period, RVI achieved the best performance, followed by PVI and WDVI.

Statistical analysis was performed for the entire dataset including 108 fields corresponding to a range of 90 to 106 observations for each date depending on the cloud cover. The period with the highest ratio was found for the period from 80 to 95 days (Table 7). The RVI showed the highest values at 80 (r = 0.72, *p* < 0.05) and 90 days (r = 0.75, *p* < 0.05) after transplanting. The NDVI performed better at 85 days (r = 0.72, *p* < 0.05), while the PVI had higher values at 95 days (r = 0.68, *p* < 0.05).

**Figure 7:**
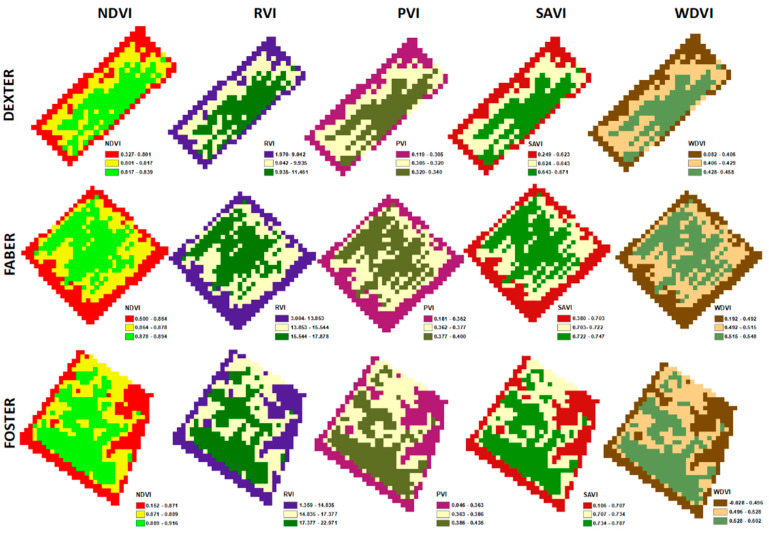
Example of the VI maps generated throughout the cultivation season at 90 days for the three varieties in quantile classification.

Apart from finding the correlations of the VIs with the yield, Sentinel-2 images can also serve as a valuable field management tool for farmers and agronomists during the cultivation season. Therefore, once the original VI layers had been cropped using the field boundaries as a mask, for each selected date, all pixels in the new raster layer were classified using a three-class quantile classification for visual interpretation of all the VIs. An example of the VI maps generated at 10 × 10 m^2^ resolution during the 90 days is shown in Figure 7.

### 3.2. Automated Machine Learning

For the AutoML experiment, the adjusted coefficient of determination (R^2^) and root mean square error (RMSE) were used to evaluate the predictive accuracy and determine the performance of the models for the best VI and period. In addition, a fivefold cross-validation was performed for each regression model to check its generalization ability and ensure its robustness. The experiments were also repeated 10 times to ensure that the final results were as accurate as possible.

Table 8 shows that the best yield predictions were made by RVI and SAVI. Specifically, these two indices reached an average R^2^ of 0.72 ± 0.02 and 0.69 ± 0.03, respectively, 90 days after transplanting. Moreover, their RMSEs were also the lowest (1.03 ± 0.03 and 1.06 ± 0.04, respectively). The remaining VIs (NDVI, WDVI, and PVI) are also among the regression models with the best performance. However, they all show a large difference relative to RVI and SAVI. Another observation from Table 8 is that the best result were achieved 90 and 85 days after transplanting.

**Figure 8:**
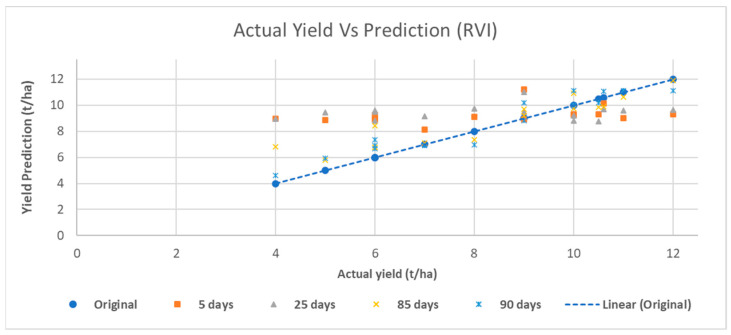
Scatter plot of actual yield vs. prediction of the four predictor dates.

Figure 8 depicts a scatter plot of RVI in which two high-performance dates (85 and 90 days after transplanting) are compared against two dates with lower performance (5 and 25 days after transplanting). It can be observed that the predictions very close to the real yield value are those in the period 85 to 95 days. On the other hand, the early dates, which have a lower performance, present predictions that deviate from the actual yield value.

**Figure 9:**
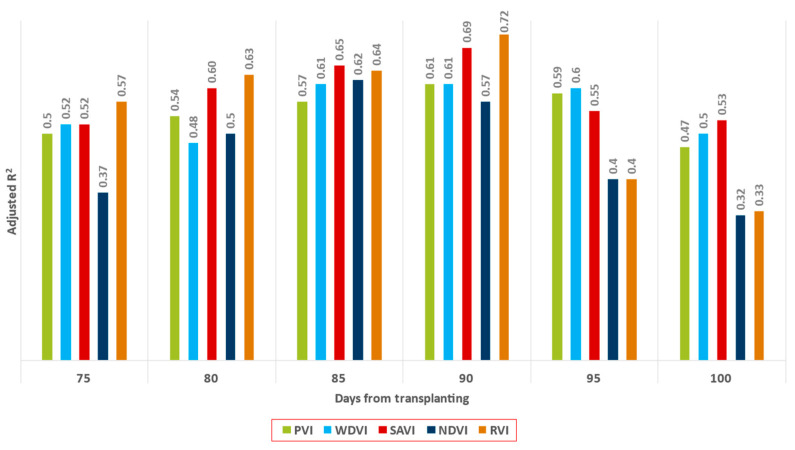
R^2^ progress along the growth period for each of the VIs.

Figure 8 shows a type of error/bias in the dataset that reveals two behaviors. When (actual) yields are less than or equal to 9 t/ha, the regressors tend to overestimate yield. On the other hand, when (actual) yields are greater than 9 t/ha, regressors tend to underestimate yield. We can hypothesize that the (ensemble) regressors tend to form a distribution with a mean/mode (apartment behavior) that acts as a gravity point in the predictions. In this particular example, this could explain yields lower than and equal to 8 t/ha and higher than 9 t/ha. In the specific case of 9 t/ha, the range of the values is not as great as for lower yields, but further research should be conducted to ensure that the predicted yield is more balanced around the real value and not prone to overestimation. Figure 9 presents the progression of adjusted R^2^ over the growing season. All indices show the best results in the 80–90-day period. This behavior is consistent with the results reported in Table 7. NDVI showed low performance overall but reached a peak in predictive power 65 days after transplanting. RVI showed the best predictive performance, but after the peak, its performance declined more rapidly than that of the other VIs. PVI and WDVI also showed low performance compared to SAVI and RVI.

**Figure 10:**
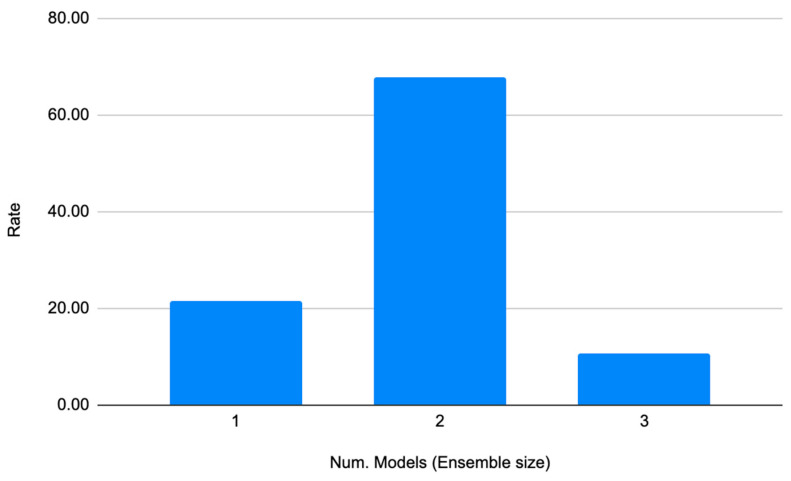
The optimal ensemble size (1, 2, 3) for the best regression models.

In addition to selecting the VIs and growth stages with the highest predictive accuracy, it was also important to examine whether using ensembles of more than one regressor was a better choice than using only one regression model. Figure 10 shows the rate of ensemble size for each of the experiments that used the VIs and growth stages shown in Table 8. This means that 500 were considered (number of rows × number of experiments × number of folds). An ensemble size of two was the preferred size (67.86%) to provide the predictions with the highest adjusted R^2^ and lowest RMSEs. The option with the second highest performance was to use single regressors (21.43%); finally, the least promising option was to use ensembles with a size of three regressors (10.71%).

Table 9 expands on Figure 10 by showing which models and ensembles achieved the best performance and how often they occurred. The combination of ARD regression and SVR was the most successful for creating an ensemble. SVR combined with Huber regression also achieved high performance. As for the individual regressors, ARD and Hubber had the highest performance several times. On the other hand, SVR was a good support in combination with other regressors but was not a successful single regressor. It is also important to note that some of the regressors evaluated by the AutoML algorithm did not show up, notably OLS regression, AdaBoost, and extra trees. When using three models to create the ensemble, the combination of ARD, random forest, and SVR was the highest-performing option.

## 4. Discussion

This study examined the performance of individual satellite-derived VIs in predicting the yield of three different varieties of processing tomato. VIs derived from spectral bands of multispectral imagery have long been used to estimate crop canopy and yield. The use of remote sensing technologies to estimate field and yield variability is becoming more common in precision agriculture due to their relatively lower cost and non-invasive approach [59]. Using Sentinel-2-like bands of other optical sensors, Veloso et al. [60] found that the results were highly correlated with fresh biomass and the green area index (GAI) and were able to detect short-lived phenological stages, enabling precise monitoring of crop development. Lykhovyd et al. [58] also found that each phenological phase has its own NDVI range. Similarly, the results of this study helped to determine the VI dynamics of tomato plants and to study plant phenology in detail. Naturally, the lowest mean values of all VIs were recorded during the period after transplanting, when canopy cover was still low and there was a lot of soil between the rows. The percentage of soil cover increases toward the middle of the season, when tomato plants reach their maximum vigor before they begin to transfer sugars to their fruit. Specifically, the highest mean VI levels were reached in July during the tomato emergence stage (75 to 95 days after planting), after which a gradual decline began. This research revealed that tomato plants exhibit a unique pattern of annual growth dynamics that is well-described and explained by VI values.

Another important point is that satellite imagery is a useful tool for estimating crop variables at the regional scale, but continuous Earth observations with high spatial resolution are often interrupted by clouds. In this study, cloud coverage did not allow for the computation of the VIs in all cases, resulting in a different number of samples for each date. For instance, the number of samples for the period of 70 to 110 days after transplanting ranged between 80 and 85 samples. At 75 and 100 days after transplanting, the sample size corresponded to 81 and 93 samples, respectively. Additionally, as usually happens when machine learning is applied to real-world data, some of the variables do not fit the Gaussian distribution. However, all the variables that fit the Gaussian distribution achieved better performances. Specifically, the range in which the VIs presented Gaussian distribution was during the period of 55 to 115 days after transplanting. A study conducted by Kaplan et al. [61] showed how this limitation can be overcome by combining observations from two publicly available space-based optical sensors (Sentinel-2 and VENµS) while simultaneously taking ground measurements over four growing seasons to monitor the evolution of tomato processing. Publicly available synthetic aperture radar imagery (SAR), particularly from Sentinel-1, has expanded the ability to monitor vegetation during day and night, even when cloud cover limits optical Earth observation. Kaplan normalized the local angle of incidence in Sentinel-1 imagery to improve estimates of leaf area index, vegetation height, and crop coefficient [62]. In addition, the Copernicus Sentinel-2 mission allows each point on Earth to be reobserved every five days with the same viewing direction, achieving even higher temporal sampling, which is particularly useful in areas with frequent cloud cover.

According to the results, the first two months reflect a weak relationship between VIs and yield due to low canopy cover. Poor correlation of all VIs was generally observed in the early growth stages of the plants, when vegetation is low and bare soil covers a significant portion of the total field area. This is explained by the fact that most of the satellite sampling grids are bare soil. In addition, the spatial resolution of 10 m imposes some limitations for crops grown in rows, as soil and vegetation between rows introduce additional noise into the spectral data. In the period from 80 to 90 days after transplanting, the predictive power of the Sentinel-2 derived VI data is satisfactory. Specifically, for NDVI, RVI, and SAVI, the Pearson coefficient consistently exceeded 0.6 for each cultivar, as well as for the entire dataset. For all cultivars, the VIs performed solidly and consistently showed r values above 0.6 at 80 days. The best performance for all VIs used in this study occurred at 90 days (r > 0.7), and the higher values of RVI during this period were considered optimal for predicting yield. Variability among the different varieties and VIs is to be expected because canopy development is a complex process and not homogeneous in all fields. Although the results are aligned with the findings of Psiroukis et al. [17], which used a similar approach, the Pearson correlation values we obtained did not reach the high values reported in their study. This discrepancy could be due to differences in the dates of the datasets used or differences in the cultivation practices employed in the Khachmaz region of Azerbaijan.

Using AutoML to find ensembles of regressors with high predictive power was one of the main goals of this work. Statistics require a model to be chosen that incorporates our knowledge of the system, and ML requires the choice of a predictive algorithm by relying on its empirical capabilities [63]. Therefore, in this study, we used a combined approach to quantify the performance of the different VIs. RVI and SAVI showed the highest correlations with the final yield at 80 (adj. R^2^ = 0.63 ± 0.02 and adj. R^2^ = 0.6 ± 0.04) to 90 days (adj. R^2^ = 0.72 ± 0.02 and adj. R^2^ = 0.69 ± 0.03), possibly indicating a critical stage when processing tomato crops undergo changes that can be distinguishable through Sentinel-2-derived data. Several researchers recommend the use of the soil-adjusted vegetation index (SAVI), which belongs to the group of spatial vegetation indices with the least bias related to soil properties and their presence on remote sensing images, allowing for better identification of plants and their differentiation from the soil [58,64]. On the other hand, NDVI underperformed in relation to the other Vis, with values below 0.62. The weak linear relationship between yield and the two indices (NDVI and PVI) may be attributed to the influence of non-weather factors determining yield, such as atmospheric influences or the fact that NDVI is sensitive to the effects of soil brightness and canopy shadow [65]. At the same time, WDVI and PVI both showed very similar performance values in terms of correlation with the final yield.

This approach is an extension of our previous work in which ensembles were not studied [29,66]. An important finding of the current work is that ensembles of two regressors achieved the highest adjusted R^2^ in most cases. This means that by averaging the predictions of two accurate regressors, precision can be increased. This result is consistent with the suggestion of Zhang [67] that the balanced use of different viewpoints from different models or regressors can lead to a more robust and consistent prediction. Consequently, the use of ensembles could be explored in other agricultural problems, such as the prediction of sugar content in grapes.

On the other hand, there is a common result with our previous work related to the high performance of some specific regressors. In particular, ARD regression and SVR achieved consistently good predictions. In contrast, tree-based regressors, such as extra trees or random forests were not successful in any of the studies. However, this should not lead to the conclusion that tree-based methods will necessarily fail in other related regression problems. Any machine-learning-based solution is subject “no free lunch” theorem [68], meaning that no algorithm is the best solution for every dataset. Therefore, even the most powerful algorithm is not suitable for all yield prediction problems. In contrast, since AutoML can be optimized by restricting the search space, using consistently successful regressors could improve the efficiency of the entire pipeline by finding a suitable solution.

## 5. Conclusions

To date, few studies have been conducted to evaluate the effectiveness of Sentinel-2 in monitoring processing tomato crop variability and predicting yield by combining statistical analysis and machine learning. In this study, an evaluation of different VIs and their relationship to phenological stages and yield in processing tomato crop was conducted. Vegetation indicators were calculated based on spectral information derived from Sentinel-2. The results show the potential of Sentinel-2 imagery to monitor field vigor and predict tomato yield at the regional level, demonstrating clear results regarding the most effective growth stages and VIs for yield prediction. The best performance for all VIs used in this study was obtained by RVI and SAVI, with the maximum accuracy and reliability occurring in the period from 80 to 90 days after transplanting. Moreover, the results show that by using AutoML, the combination of ARD regression and SVR was the combination of regressors with the highest predictive accuracy.

The findings presented in this paper are encouraging for the development of a large-scale monitoring system, especially based on the Sentinel-2 mission, which provides free access to data with high spatial and temporal resolution from most regions of the world. The pattern of annual NDVI change in tomato crops could be integrated into models for automatic crop identification, mapping, and phenological monitoring.

## Figures and Tables

**Table 1:** Acquisition dates of satellite data for the 2021 growing season.

Month	Dates						
April	Cloud cover	Cloud cover					
May	03/05/21	13/05/21	18/05/21	23/05/21	28/05/21		
June	02/06/21	07/06/21	12/06/21	17/06/21	22/06/21	27/06/21	
July	02/07/21	07/07/21	12/07/21	17/07/21	22/07/21	27/07/21	
August	0/08/2021	06/08/21	11/08/21	16/08/21	21/08/21	26/08/21	31/08/21
September	Cloud cover	10/09/21	15/09/21				

**Table 2:** The selected VIs used in this study and their respective spectral equations.

Index	Equation	Reference
NDVI	$\frac{\left(NIR-RED\right)}{\left(NIR+RED\right)}$	Rouse et al. [30]
WDVI	*NIR* − *S* ∗ RED where *S* is the slope of the soil line from a plot of red versus near-infrared.	Clevers [31,32]
PVI	(NIR−a∗RED−b)(a^2+1) where *a* is the slope of the ground line, and *b* is the ground line’s gradient.	Richardson & Wiegand [33]
RVI	NIR/RED	Pearson & Miller [34]
SAVI	$(\frac{\left(NIR-RED\right)}{\left(NIR+RED+L\right)})*\left(1+L\right)$ where *L* is a soil adjustment factor	Huete [35]

**Table 3:** Descriptive statistics of the yields (t/ha) corresponding to Dexter, Faber, and Foster hybrids.

Yield	Mean	Min	Max	SD	CV (%)
Dexter	98.20	55.00	135.00	21.05	21.44
Faber	90.05	65.00	120.00	18.40	20.42
Foster	86.68	40.00	124.00	21.79	25.13
Total	93.91	40.00	135.00	21.20	22.57

**Table 4:** The Pearson coefficient representing the relationships between the derived VIs and the yield of the Dexter hybrid.

VI	Pearson Coefficient			
	80 Days	85 Days	90 Days	95 Days
NDVI	0.70 *	**0.72** *	0.68 *	0.65 *
RVI	**0.71** *	0.71 *	**0.75** *	0.60 *
SAVI	0.68 *	0.68 *	0.74 *	0.65 *
PVI	0.67 *	0.67 *	**0.75** *	0.71 *
WDVI	0.58 *	0.66 *	0.75 *	**0.72** *

* Correlation is significant at the 0.05 level.

**Table 5:** The Pearson coefficient representing the relationships between the derived VIs and the yield of the Faber hybrid.

VI	Pearson Coefficient			
	75 Days	80 Days	85 Days	90 Days
NDVI	**0.82** *	0.71 *	**0.85** *	0.81 *
RVI	0.80 *	0.75 *	0.70 *	0.86 *
SAVI	0.79 *	0.78 *	0.81 *	**0.90** *
PVI	0.63 *	**0.82** *	0.80 *	0.83 *
WDVI	0.58 *	0.79 *	0.79 *	0.87 *

* Correlation is significant at the 0.05 level.

**Table 6:** The Pearson coefficient representing the relationships between the derived VIs and the yield of the Foster hybrid.

VI	Pearson Coefficient			
	75 Days	80 Days	85 Days	90 Days
NDVI	0.59 *	0.64 *	0.68 *	0.75 *
RVI	**0.77** *	**0.74** *	**0.72** *	**0.84** *
SAVI	0.70 *	0.71 *	0.66 *	0.79 *
PVI	0.72 *	0.72 *	0.64 *	0.77 *
WDVI	0.71 *	0.72 *	0.66 *	0.78 *

* Correlation is significant at the 0.05 level.

**Table 7:** The Pearson coefficient representing the relationships between the derived VIs and the yield of all hybrids.

VI	Pearson Coefficient			
	80 Days	85 Days	90 Days	95 Days
NDVI	0.68 *	**0.72** *	0.70 *	0.63 *
RVI	**0.72** *	0.70 *	**0.75** *	0.56 *
SAVI	0.68 *	0.69 *	0.74 *	0.65 *
PVI	0.67 *	0.64 *	0.72 *	**0.68** *
WDVI	0.58 *	0.65 *	0.73 *	0.69 *

* Correlation is significant at the 0.05 level.

**Table 8:** The 10 best-performing vegetation indices and periods.

VI	Period (Days)	Adjusted R^2^	RMSE
RVI	90	0.72 ± 0.02	1.03 ± 0.03
SAVI	90	0.69 ± 0.03	1.06 ± 0.04
SAVI	85	0.65 ± 0.03	1.09 ± 0.03
RVI	85	0.64 ± 0.02	1.12 ± 0.06
RVI	80	0.63 ± 0.02	1.13 ± 0.04
NDVI	85	0.62 ± 0.04	1.14 ± 0.06
WDVI	90	0.61 ± 0.02	1.15 ± 0.03
WDVI	85	0.61 ± 0.03	1.15 ± 0.04
PVI	90	0.61 ± 0.05	1.16 ± 0.05
SAVI	80	0.60 ± 0.04	1.15 ± 0.05

**Table 9:** The 10 best-performing models (ensembles and single regressors).

Ensemble/Model	R^2^	Num. Appearances.
ARD Regr. + SVR	0.67 ± 0.02	109
ARD Regr.	0.65 ± 0.03	87
Huber Regr. + SVR	0.65 ± 0.02	74
Huber Regr.	0.65 ± 0.04	63
ARD Regr. + Huber Regr.	0.66 ± 0.03	52
ARD Regr. + Random Forest + SVR	0.63 ± 0.03	41
ARD Regr. + Decision Tree	0.65 ± 0.02	30
Huber Regr. + Theil-Sen Regr.	0.64 ± 0.04	23
SVR + Theil-Sen Regr.	0.65 ± 0.03	12
ARD Regr. + Random Forest	0.63 ± 0.05	5

## Data Availability

The data used in this manuscript are open Sentinel-2 data, which can be accessed through ESA’s Copernicus Hub. The ground truth data used in this study were collected by farmers and agronomists and are available upon request from the corresponding author. The data are not publicly available due to a privacy agreement with Corteva Agriscience Hellas.
